# The Potential Role of hsa_circ_0005505 in the Rupture of Human Intracranial Aneurysm

**DOI:** 10.3389/fmolb.2021.670691

**Published:** 2021-07-14

**Authors:** Xin Chen, Shuzhe Yang, Junhua Yang, Qingyuan Liu, Maogui Li, Jun Wu, Hao Wang, Shuo Wang

**Affiliations:** ^1^ Department of Neurosurgery, Beijing Tiantan Hospital, Capital Medical University, Beijing, China; ^2^ China National Clinical Research Center for Neurological Diseases, Beijing, China; ^3^ Center of Stroke, Beijing Institute for Brain Disorders, Beijing, China; ^4^ Beijing Key Laboratory of Translational Medicine for Cerebrovascular Diseases, Beijing, China

**Keywords:** circular RNA, intracranial aneurysm, vascular smooth muscle cell, phenotype modulation, protein kinase C

## Abstract

**Objective:** Recently, abundant number of studies have revealed many functions of circular RNAs in multiple diseases, however, the role of circular RNA in the rupture of human intracranial aneurysm is still unknown. This study aims to explore the potential functions of circular RNA in the rupture of human intracranial aneurysms.

**Methods:** The differentially expressed circular RNAs between un-ruptured intracranial aneurysms (*n* = 5) and ruptured intracranial aneurysms (*n* = 5) were analyzed with the Arraystar human circRNAs microarray. Quantitative real-time PCR (qPCR) was used to verify the results of the circRNA microarray. The role of circular RNA in intracranial aneurysm rupture was assessed *in vitro*. MTT assay, CCK-8 assay, Caspase3/7 assay, assay of cell apoptosis and Celigo wound healing was conducted to evaluate the relationship between circular RNA and the rupture of human intracranial aneurysms.

**Results:** A total of 13,175 circRNA genes were detected. Among them 63 circRNAs upregulated and 54 circRNAs downregulated significantly in ruptured intracranial aneurysms compared with un-ruptured intracranial aneurysms (*p* < 0.05 Fold Change > 1.5). Five upregulated circRNAs were selected for further study (hsa_circ_0001947, hsa_circ_0043001, hsa_circ_0064557, hsa_circ_0058514, hsa_circ_0005505). The results of qPCR showed only hsa_circ_0005505 significantly upregulated (*p* < 0.05). The expression of hsa_circ_0005505 was higher in ruptured intracranial aneurysm tissues. And our *in vitro* data showed that hsa_circRNA_005505 promotes the proliferation, migration and suppresses the apoptosis of vascular smooth muscle cell.

**Conclusion:** This study revealed an important role of hsa_circ_0005505 in the proliferation, migration and apoptosis of vascular smooth muscle cell, and indicated that hsa_circ_0005505 may associate with the pathological process of intracranial aneurysms.

## Introduction

Intracranial aneurysm (IA) is a cerebrovascular disorder characterized by a regional ballooning of intracranial arteries. The incidence of intracranial aneurysm in general population is 1.8–8.4% (Kang et al., 2015). Rupture of an intracranial aneurysm can lead to fatal subarachnoid hemorrhage (SAH) which carries a high risk of death or disability. How to assess the possibility of intracranial aneurysm rupture is still inconclusive. In recent 5 years, plenty of researchers studied about the molecular mechanism of the intracranial aneurysm rupture. Many biological processes such as inflammation, the phenotypic changes of vascular smooth muscle cell (VSMC), cell adhesion, atherosclerosis and abnormal extracellular matrix metabolism may participate in the mechanism of intracranial aneurysm rupture (Jiang et al., 2018; Jabbarli et al., 2020). Existing evidence suggests that the process of abnormal inflammatory response in blood vessel wall is initiated by hemodynamic changes, induces the activation of signaling pathways such as NFκB through unknown mechanism. Then, Matrix Metalloproteinases (MMP) such as MMP2 and MMP9 generates the extracellular matrix degradation and phenotypic changes of VSMC which leads to the formation and rupture of aneurysm (Cheng and Wang, 2013; Liu et al., 2018; Lai et al., 2019). VSMC distributes in the middle layer of artery wall and is the main cell that synthesizes matrix of artery wall. Following a number of stimuli, the phenotype of VSMC can switch from a contractile type to a pro-inflammatory, dedifferentiated phenotype characterized by the increased expression of OPN (osteopontin), YAP1 (Yes-associated protein 1), inflammatory factors and MMP which may be crucial to aneurysm formation and rupture (Yoshida and Owens, 2005). During the VSMC phenotype change, the proliferation, migration and synthetic ability of VSMC will be promoted and are major events leading to the intracranial aneurysm rupture (Chalouhi et al., 2012).

Circle RNAs (termed circRNAs) are one type of non-coding RNAs (ncRNAs) that are formed covalently closed loop structures and widely expressed in human cells (Salzman et al., 2012; Li et al., 2015). In recent years, as the widespread use of RNA sequencing technique and biological experiments, many circRNAs have been discovered and proved to be highly expressed in a tissue-specific or cell type-specific manner (Liang and Wilusz, 2014; Starke et al., 2015). And some researchers have revealed the biological function of circRNA. It was reported that circRNAs can function as miRNA sponges that control gene transcription (Hansen et al., 2013), besides, circRNAs can interact with different proteins and affect the function of combined protein (Du et al., 2017). Moreover, circRNAs have the function of encoding functional peptides or proteins (Pamudurti et al., 2017; Yang et al., 2017). Emerging evidence also suggests a potential role of circRNAs in different human diseases (Chen et al., 2016). However, research about the role of circRNAs in the formation and rupture of intracranial aneurysm are rare, and the overall pathophysiological contributions of circRNAs to intracranial aneurysm remain largely unknown.

In this study we acquired differentially expressed circRNAs between ruptured intracranial aneurysm (RIA) tissues and un-ruptured intracranial aneurysm (UIA) tissues through circRNA microarray. We further detected hsa_circ_0005505 and assessed the role of hsa_circ_0005505 in intracranial aneurysm rupture *in vitro*.

## Methods

### Patients and Specimens

A total of 10 pairs of ruptured and un-ruptured intracranial aneurysm tissues were obtained from surgical resection during the aneurysm clipping surgery in Beijing Tiantan Hospital. The collection of human specimens was approved by the Medical Ethics Committee of Beijing Tiantan Hospital, Capital Medical University. Written informed consent was obtained from each patient according to the policies of the committee. All specimens were stored in liquid nitrogen, and five pairs of samples were used to conduct circRNA microarray analysis, other samples were used to perform qPCR.

### Total RNA Isolation and Quality Control

We extracted RNA with the use of Trizol Reagent (Invitrogrn, NY, United States) from five paired ruptured and un-ruptured intracranial aneurysms according to the manufacturer’s instructions. The quality and concentration of RNA was tested by the NanoDrop ND- 1000 (Thermo Fisher Scientific, Wilmington, United States) (Supplementary Table S1).

### RNA Labeling and Hybridization

Sample labeling and array hybridization were performed according to the manufacturer’s protocol (Arraystar, Rockville, United States). Briefly, total RNAs were digested with Rnase R (Lucigen, Middleton, United States) to remove linear RNAs and rich circular RNAs. Then, the enriched circular RNAs were amplified and transcribed into fluorescent cRNA utilizing a random priming method (Arraystar Super RNA Labeling Kit; Arraystar, Rockville, United States). The labeled cRNAs (Supplementary Table S2) were purified by RNeasy Mini Kit (Qiagen, Duesseldorf, Germany). The concentration and specific activity of the labeled cRNAs (pmol Cy3/μg cRNA) were measured by NanoDrop ND-1000 (Thermo Fisher Scientific, Wilmington, United States). 1 μg of each labeled cRNA was fragmented by adding 5 μl 10 × blocking agent and 1 μl of 25 × fragmentation buffer, then heated the mixture at 60°C for 30 min, finally 25 μl 2 × hybridization buffer was added to dilute the labeled cRNA. 50 μl of hybridization solution was dispensed into the gasket slide and assembled to the circRNA expression microarray slide. The slides were incubated for 17 h at 65°C in an Agilent Hybridization Oven (Santa Clara, United States). The hybridized arrays were washed, fixed and scanned using the Agilent Scanner G2505C (Santa Clara, United States).

### CircRNA Microarray Analysis

Agilent Feature Extraction software (version 11.0.1.1) was used to analyze acquired array images. Quantile normalization and subsequent data processing were performed using the R software limma package. Differentially expressed circRNAs with statistical significance between two groups were identified through Volcano Plot filtering. Differentially expressed circRNAs between two samples were identified through Fold Change filtering. Hierarchical Clustering was performed to show the distinguishable circRNAs expression pattern among samples.

### Real-Time Quantitative PCR

Real-time PCR was used to verify differentially expressed circRNAs obtained from microarray analysis. RNase R (Lucigen, Middleton, United States) was used to purify the circRNAs again. The relevant cDNAs were composed (M-MLV, promega, Madison, United States) and stored in −20°C. QuantStudio5 Real-time PCR System (Applied Biosystems, Waltham, United States) was used to perform qPCR. The sequence of circRNA results was acquired from the database “circBase” (http://circrna.org). Primers were produced by RiboBio (Guangzhou, China) (Supplementary Table S3). Because of the influence of concentration quantitative error and reverse transcription efficiency error, the cDNA content of every sample was different. In order to correct these errors, we regarded housekeeping gene β-actin as internal reference, as a result, we accepted the ratio of genes to be tested and internal reference, in other words, the relative content of the gene to be tested. All qPCR experiments in our research were performed triplicate.

### Construction of circRNA-miRNA-mRNA Network

CSCD (http://gb.whu.edu.cn/CSCD/) was used to recognize miRNAs binding on our target circRNA. Three algorithms including Targetscan (Lewis et al., 2003), miRDB (Wong and Wang, 2015), and miRTarBase (Hsu et al., 2011) were used to analyze parental genes of miRNAs binding on the target circRNA. The circRNA-miRNA-mRNA network was visualized by Cytoscape (version3.7.1).

### Gene Ontology and Kyoto Encyclopedia of Genes and Genomes Pathway Analysis

We assumed that our target circRNA may have molecular interactions with these genes, or our target circRNA may regulate biological functions through these genes. GO analysis on genes correlated with these miRNAs was performed by DAVID (https://david.ncifcrf.gov/). The *p* value after adjustment represents the significance of GO terms. We also perform KEGG pathway analysis of parental genes of circRNA-binding miRNAs, in order to reveal the biological or pathological processes which circRNAs participate in. The *p* value after adjustment represents the significance of pathway correlations as well.

### Cell Culture

Human brain vascular smooth muscle cells (HBVSMCs) were obtained from Bnbio (BNCC102172, Beijing, China). The cells were cultured in Smooth Muscle Cell Medium (SMCM, Sciencell, San Diego, United States) containing 2% fetal bovine serum (FBS, Sciencell, San Diego, United States), 5 ml of smooth muscle cell growth supplement (SMCGS, Sciencell, San Diego, United States), and 5 ml of penicillin/streptomycin solution (P/S, Sciencell, San Diego, United States) at 37°C in an incubator of 5% CO2.

### Transduction of Cells

The hsa_circ_0005505 specific shRNA, their relevant lentiviruses [LV-circRNA-RNAi (74402-1), LV-circRNA-RNAi (74403-2), LV-circRNA-RNAi (74404-1)] and negative control lentivirus were obtained from Shanghai Genechem Co., LTD. (Shanghai, China). HBVSMCs were transduced with individual types of lentivirus at a multiplicity of infection (MOI) of 50 and the ideal value of infection efficiency was 80%.

### MTT Assay

MTT assay was used to measure the viability of HBVSMCs according to the manufacturer’s instructions (Dingguo Biotech, Shanghai, China). HBVSMCs (2 × 10^3^ per well) were plate in 96-well plates and treated with 20 μl of 5 mg/ml MTT solution, then the spectrophotometrically at 490 nm was analyzed by automatic microplate reader (Tecan infinite, Mannedorf, Switzerland). MTT assay was performed triplicate in our research.

### Cell Counting Kit-8 Assay

CCK-8 assay was utilized to test the cell viability in order to verify the effect of the target gene on cell proliferation. The assay was performed according to manufacturer’s instructions (Dojindo Laboratories, Kumamoto, Japan). HBVSMCs (2 × 10^3^ per well) were plate in 96-well plates and treated with 10 μl of CCK-8 solution, then the spectrophotometrically at 450 nm was analyzed by automatic microplate reader (Tecan infinite, Mannedorf, Switzerland). CCK-8 assay was performed triplicate.

### Apoptosis Analysis

Cell apoptosis was analyzed in two ways. The caspase3/7 assay was used to verify the effect of the target gene on apoptosis of cells by using the Caspase-Glo 3/7 Assay reagent test kit (Promega, Madison, United States). The apoptosis of cells was analyzed after 3 days since infection. We also analyzed cell apoptosis by using the Annexin V-APC Apoptosis Detection Kit (eBiosciences, San Diego, United States) according to the manufacturer’s instruction. HBVSMCs were stained with APC and then analyzed by fluorescence-activated cell sorting using FACScan (BD Biosciences, NYC, United States) after 5 days since infection. Both apoptosis analyses were performed three times.

### Wound-Healing Assay

The wound -healing assay was used to evaluate the migration rate of cells. The transfected HBVSMCs were seeded into 96 well plates (5 × 10^4^ per well). After 24 h incubation, parallel wounds with similar width were made in each well by 96 Wounding Replicator (VP scientific, United States). Wound closure level was monitored by Celigo (Nexcelom, Boston, United States) in 0, 8, and 24 h after wounded and lastly analyze the migration rate. Wound-healing assay was performed triplicate.

### Western Blot

Protein was isolated from HBVSMC using a RIPA lysis buffer (Aspen biological, Wuhan, China). The protein content was assessed by using a BCA protein assay kit (Aspen biological, Wuhan, China). Protein lysates (40 μg/sample) were separated on 10% SDS-PAGE and transferred to polyvinylidene difluoride membranes (PVDF, Millipore, Billerica, United States). Then membranes were probed with following primary antibodies: anti-YAP1 (cat. no. ab205270, 1:1000, Abcam, Cambridge, United States), anti-MMP2 (cat. no. ab92536, 1:1000, Abcam, Cambridge, United States), anti-MMP9 (cat. no. ab76003, 1:500, Abcam, Cambridge, United States), anti-OPN (cat. no. ab214050, 1:1000, Abcam, Cambridge, United States) and anti-GAPDH (cat. no. ab37168, 1:10000, Abcam, Cambridge, United States), then incubated overnight at 4°C. After that, horseradish peroxidase-conjugated goat anti-mouse immunoglobulin G (IgG, cat. no. as1106; 1:10000; Aspen biological, Wuhan, China) and horseradish peroxidase-conjugated goat anti-rabbit immunoglobulin G (IgG, cat. no. as1107; 1:10000; Aspen biological, Wuhan, China) were used to detect protein band at room temperature for 30 min. Signals were detected using an enhanced chemiluminescence kit (ECL kit, Aspen biological, Wuhan, China) according to manufacturers’ instruction. The band density was quantified with the AlphaEaseFC software. GAPDH served as the loading control. Each experiment was performed at least three times.

### Statistical Analysis

The fold-changes were estimated by unpaired Student’s *t*-test and used to identify the differentially expressed circRNAs in the sample of intracranial aneurysms. CircRNAs were selected as differentially expressed with a *p* < 0.05 and a fold-change > 1.5, which means they were statistically significant. The significance of qRT-PCR was assessed by Student’s *t*-test and *p* < 0.05 was considered statistically significant, it was analyzed by GraphPad Prism 8.4.0 (GraphPad Software, La Jolla, CA, United States). Other statistical methods such as chi-squared test, Wilcoxon signed-rank test and Mann Whitney U test were also performed. All statistical analyses were performed by SPSS 19.0 (SPSS, Inc., Chicago, United States).

## Results

### Identification of circRNA Microarray in Human UIA and RIA Samples

**FIGURE 1:**
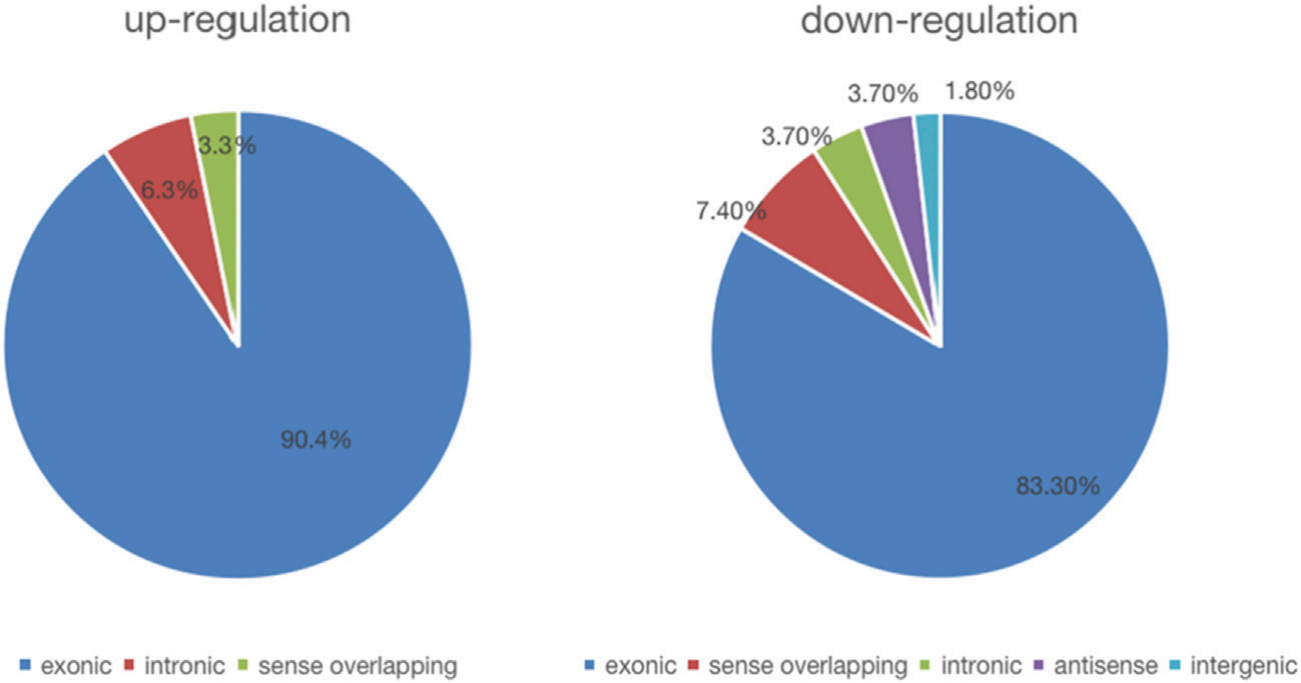
The constituent of dysregulated circRNAs.

**FIGURE 2:**
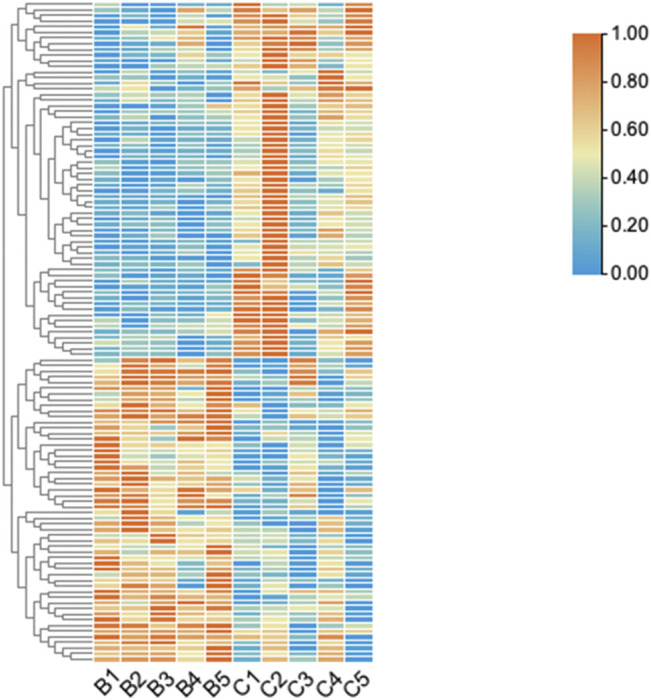
The hierarchical clustering of differentially expressed circRNAs. “Red” indicates high relative expression, and “green” indicates low relative expression. Group B represented unruptured intracranial aneurysm samples, group C represented ruptured intracranial aneurysm samples.

**FIGURE 3:**
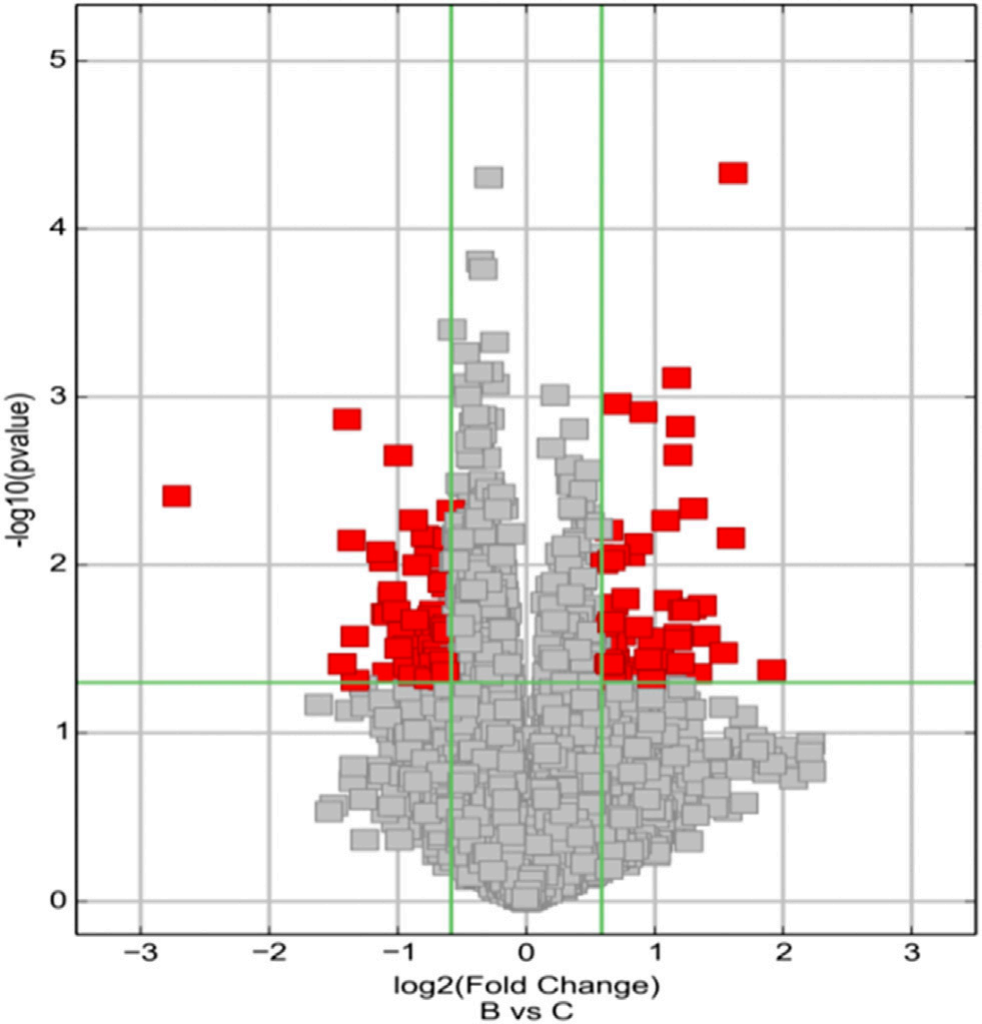
Volcano Plots are used for visualizing differential expression between two different conditions. The vertical lines correspond to 1.5-fold up and down, respectively, and the horizontal line represents a *p*-value of 0.05. So, the red point in the plot represents the differentially expressed circRNAs with statistical significance. Group B represented unruptured intracranial aneurysm samples, group C represented ruptured intracranial aneurysm samples.

We detected a total of 13,174 circRNAs by Arraystar human circRNA Microarray. Among them, 117 circRNAs dysregulated between UIA and RIA tissues (fold-change > 1.5, *p* < 0.05) (Supplementary Table S4) and 93 of them have been identified by other studies before. Comparing RIA with UIA tissues, 63 circRNAs upregulated while 54 circRNAs downregulated. All circRNAs that we detected contain five types: “exonic,” “intronic,” “antisense,” “sense overlapping” and “intergenic.” In the 63 upregulated circRNAs, 57 (90.4%) circRNAs arising from the exons of the linear transcript, 4 (6.3%) circRNAs arising from the introns of the linear transcript, and 2 (3.3%) circRNAs transcribed from same gene locus as the linear transcript, but not classified into “exonic” and “intronic” which were classified into “sense overlapping” (Figure 1). However, in 54 downregulated circRNAs, 45 (83.3%) circRNAs were arising from exons, 4 (7.4%) circRNAs were classified into “sense overlapping,” 2 (3.7%) circRNAs were “intronic,” 2 (3.7%) circRNAs whose gene locus overlap with the linear RNA but transcribed from the opposite strand which means the “antisense,” and 1 (1.8%) circRNA was “intergenic” which means located outside known gene locus (Figure 2). The variation of circRNA expression between the UIA and RIA samples were assessed (Figure 3). Five upregulated circRNAs were selected for further investigation (hsa_circ_0001947, hsa_circ_0043001, hsa_circ_0064557, hsa_circ_0058514, hsa_circ_0005505). Besides fold-change > 1.5 and *p* < 0.05, all these five circRNAs’ raw intensities in UIA and RIA groups were more than 200 and their parental genes were well investigated by other studies in order to reveal these circRNA’s functions better.

**FIGURE 4:**
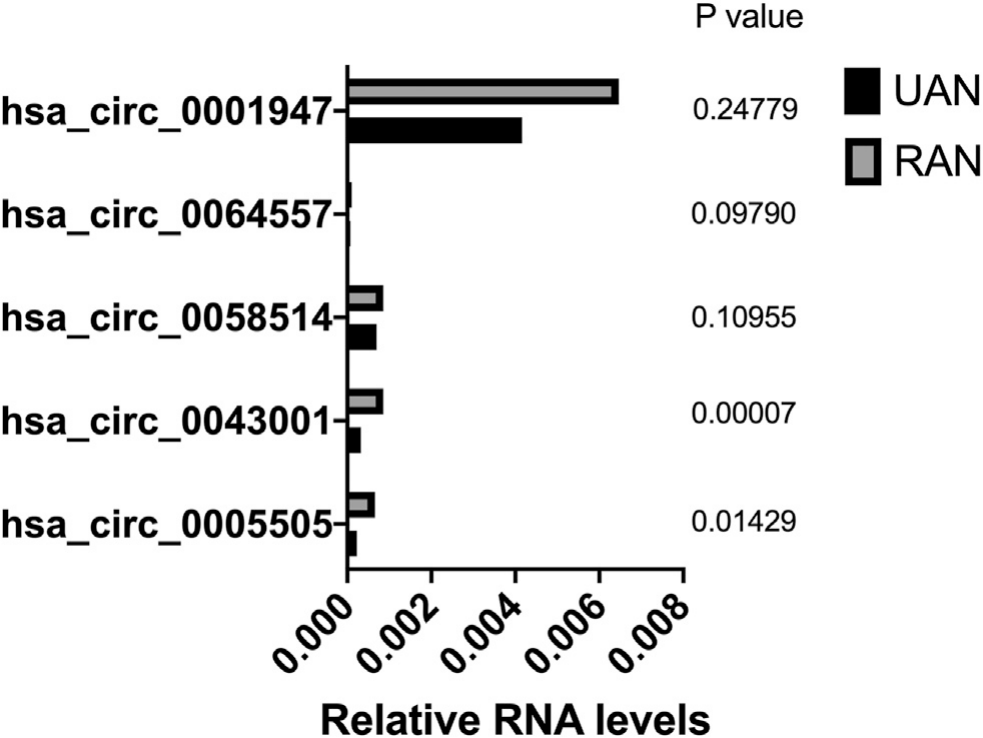
Validation of the differential expression of five upregulated circRNAs.

The circRNA microarray profiling expression results were verified through quantitative reverse transcription PCR (qPCR) in five paired UIA and RIA samples. All these five selected circRNAs were upregulated which in agreement with the microarray results, but only hsa_circ_0005505 and hsa_circ_0043001 upregulated significantly (*p* < 0.05) (Figure 4). The parental gene of hsa_circ_0005505 is interleukin 1 receptor associated kinase 3 (IRAK3) and ras homolog family member T1 (RHOT1) is the parental gene of hsa_circ_0043001. Compared with RHOT1, IRAK3 has been revealed to be related with MAPK and NF-kappa B signaling pathway (Ge et al., 2019; Wu et al., 2020), which participate in the pathological process of intracranial aneurysm. As a result, hsa_circ_0005505 was finally selected for further research.

### Characteristics and Functions of hsa_circ_0005505

**FIGURE 5:**
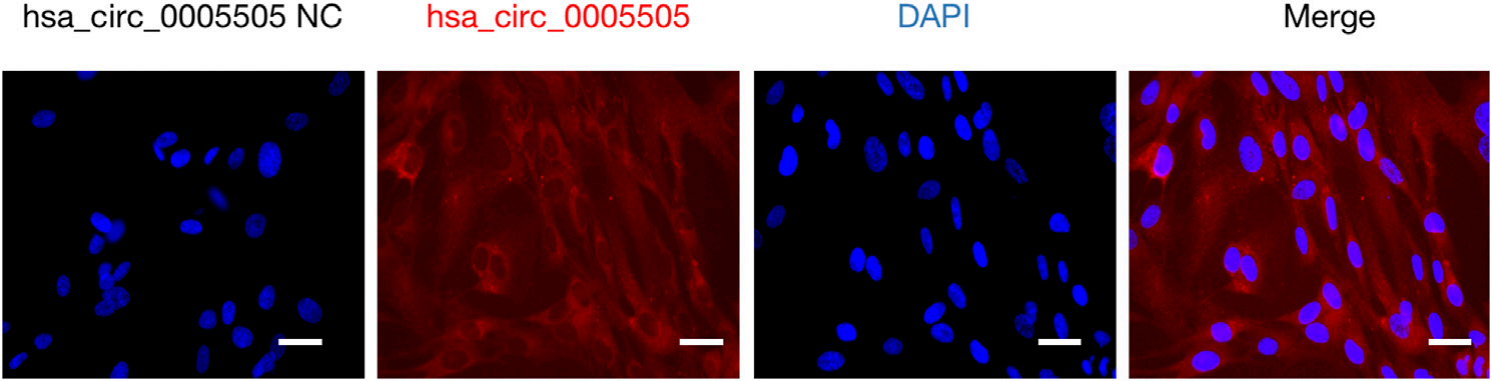
Fluorescence *in situ* hybridization assay showing cytoplasmic localization of hsa_circ_0005505 in HBVSMCs. Scale bar = 25 μmm. NC = negative control.

**FIGURE 6:**
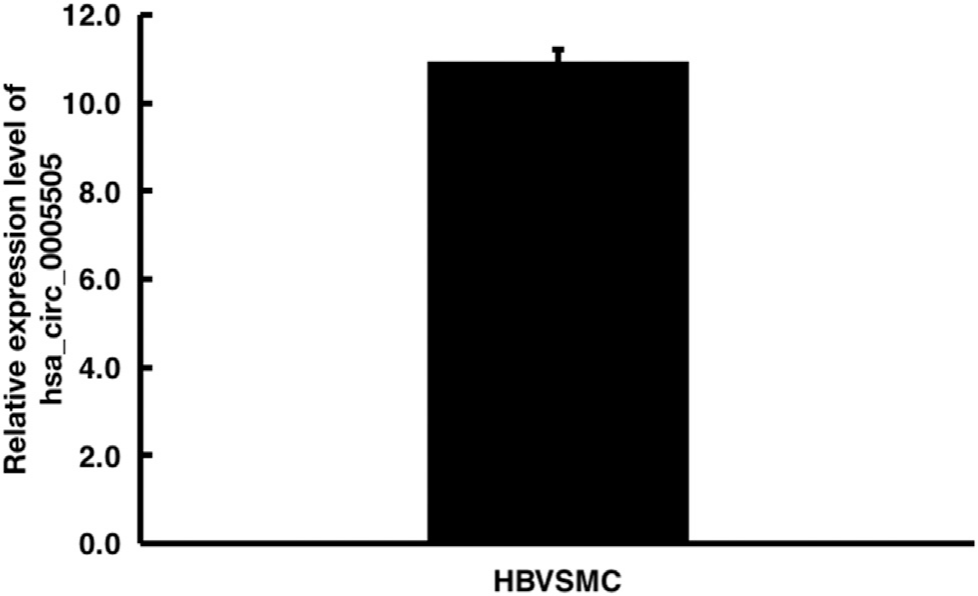
Quantitative real-time PCR analysis of hsa_circ_0005505 expression level in HBVSMCs. GAPDH was regarded as the reference gene. △Ct value = Ct value of target gene - Ct value of reference gene. △Ct value ≤ 12 represents the high expression of target gene in cells, △Ct value ≥ 16 represents the low expression of target gene in cells.

The hsa_circ_0005505 (chr12:66597490-66622150) is partly derived from exon 5–11 in human endogenous IRAK3. The genomic sequence of hsa_circ_0005505 is 24660nt and the spliced length is 754nt. To explore the potential functions of hsa_circ_0005505, we found hsa_circ_0005505 contain 14 open reading frames (ORFs) through ORFfinder (https://www.ncbi.nlm.nih.gov/orffinde) (Table 1), but only the longest one has the ability to encode protein. The result of the Conserved Domains (https://www.ncbi.nlm.nih.gov/Structure/cdd/wrpsb.cgi) showed that, the ORF of hsa_circ_0005505 may encode PKC (Protein Kinases, catalytic domain) _like super family (Bit Score = 260.20, E-value = 4.62e-88). Besides encoding proteins, the other potential function of circRNA is microRNA (miRNA) sponges. Through CSCD, miRNAs binding on hsa_circ_0005505 have been found (Supplementary Table S5). Also, we recognized proteins binding on hsa_circ_0005505 from CSCD, and the result was listed in Table 2. Furthermore, fluorescence *in situ* hybridization (FISH) against hsa_circ_0005505 showed the predominant cytoplasmic distribution of hsa_circ_0005505 (Figure 5). We also investigated the expression level of hsa_circ_0005505 in HBVSMCs. According to the result of qPCR, hsa_circ_0005505 is significantly upregulated in HBVSMCs (Figure 6).

**TABLE 1:** Open reading frames that detected from hsa_circ_0005505.

Label	Strand	Frame	Start	Stop	Length (nt|aa)	Nucleotide sequence
ORF1	+	1	259	345	87|28	MSPWIMFLFLNIMKKEYCLNLPSAFKIS
ORF2	+	1	433	543	111|36	MLSNYLNRRKKCSVRSIGRGFYLSLKFYYCFITQTY
ORF3	+	2	656	>754	99|32	MAHSNRYINRNIQSHSLPAQRSTMLGHLWQYIK
ORF4	+	3	24	>752	729|242	MDVRHIEKYVDQGKSGTRELLWSWAQKNKTIGDLLQVLQEMGHRRAIHLI
						TNYGAVLSPSEKSYQEGGFPNILFKETANVTVDNVLIPEHNEKGILLKSS
						ISFQNIIEGTRNFHKDFLIGEGEIFEVYRVEIQNLTYAVKLFKQEKKMQC
						KKHWKRFLSELEVLLLFHHPNILELAAYFTETEKFCLIYPYMRNGTLFDR
						LQCVGDTAPLPWHIRIGILIGISKAIHYLHNVQPCSVICGSIS
ORF5	—	1	700	587	114|37	MALDIPINIPIRMCQGSGAVSPTHCNLSKSVPFLMYG
ORF6	—	1	409	332	78|25	MYTSKISPSPIRKSLWKFLVPSMIF
ORF7	—	1	328	215	114|37	MMEDLSSIPFSLCSGIRTLSTVTLAVSLNNIFGNPPS
ORF8	—	1	115	35	81|26	MVLFFCAQDQSNSLVPLLPWSTYFSI
ORF9	—	2	726	559	168|55	MVERCAGSEWLWIFLLIYRFECAKGVGPCHLHTAICQKVFHFSCMDKSDRTSQSL
ORF10	—	2	555	448	108|35	MQPTLVCLGDETVVKLQAQIKTSSNASYTAFFSPV
ORF11	—	2	432	352	81|26	MLGFESPLCIPQKSLLLQLGSLCGNF
ORF12	—	2	75	>1	75|24	MFHFYLGLHTFQYDEHPASCLKVSL
ORF13	—	3	629	540	90|29	MQSVKKCSISHVWINQTELLSLCKICSQL
ORF14	—	3	335	240	96|31	MKADGRFKQYSFFIMFRNKNIIHGDIGCFLE

ORF: open reading frame.

**TABLE 2:** Proteins binding on hsa_circ_0005505.

ID	RBP	Start	End	Details
1	FUS_Human_GSE43308_HITS-CLIP	66605210	66605225	HHMF2_86273_FUS_rep2_1
2	FUS_Human_GSE43308_HITS-CLIP	66604094	66604110	HHMF2_86272_FUS_rep2_2
3	FUS_Human_GSE43308_HITS-CLIP	66621697	66621715	HHMF2_86276_FUS_rep2_4
4	DGCR8_Human_GSE39086_HITS-CLIP	66608982	66609004	HHCT1_6374_DGCR8_T7.1_1
5	FUS_Human_GSE43308_HITS-CLIP	66619959	66619978	HHMF2_86275_FUS_rep2_1
6	FUS_Human_GSE43308_HITS-CLIP	66603282	66603300	HHMF3_96612_FUS_rep3_3
7	FUS_Human_GSE43308_HITS-CLIP	66611178	66611193	HHMF3_96617_FUS_rep3_1
8	FUS_Human_GSE43308_HITS-CLIP	66604013	66604032	HHMF3_96613_FUS_rep3_3
9	FUS_Human_GSE43308_HITS-CLIP	66609043	66609058	HHMF3_96616_FUS_rep3_1
10	FUS_Human_GSE43308_HITS-CLIP	66606215	66606247	HHMF3_96615_FUS_rep3_3
11	FUS_Human_GSE43308_HITS-CLIP	66604755	66604771	HHMF3_96614_FUS_rep3_3
12	DGCR8_Human_GSE39086_HITS-CLIP	66618821	66618842	HHCD1_11057_DGCR8_D8.1_1

RBP: RNA Binding Proteins.

### Construction of circRNA-miRNA-mRNA Network and GO, KEGG Pathway Analysis

**FIGURE 7:**
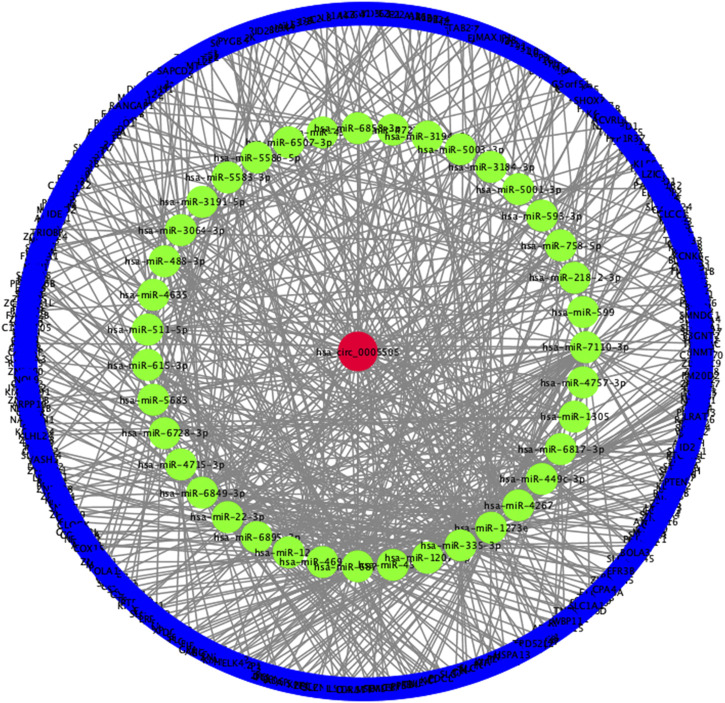
Constructed hsa_circ_0005505−miRNA−mRNA network. Red ellipse: hsa_circ_0005505, green ellipse: miRNAs potentially interacted with hsa_circ_0005505, blue ellipse: genes positively co-expressed with hsa_circ_0005505.

**FIGURE 8:**
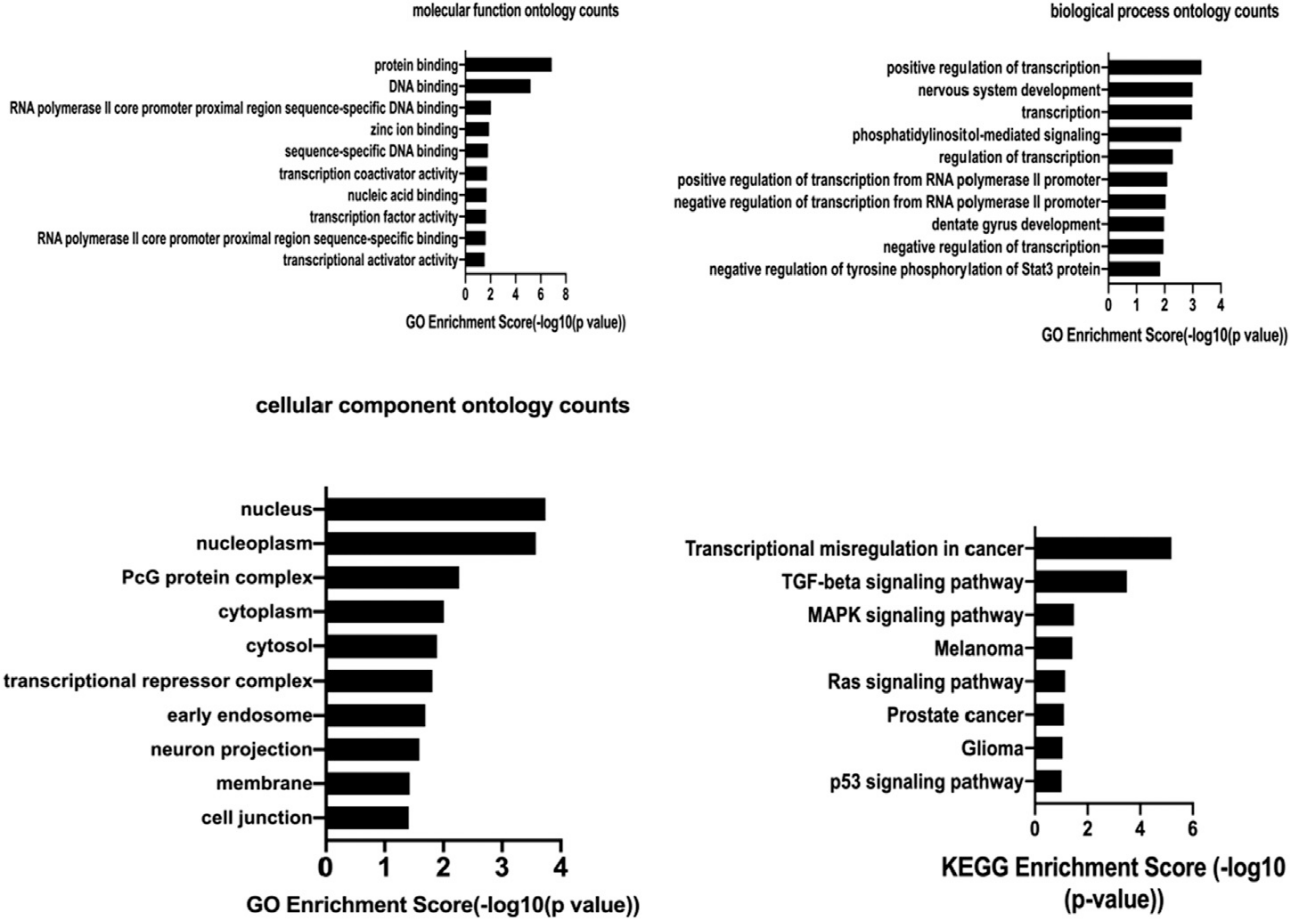
The molecular function ontology analysis, biological process ontology analysis,cellular component ontology analysis and KEGG pathway analysis of hsa_circ_0005505. GO enrichment score and KEGG enrichment score represent the number of genes enriched in the cluster.

Based on the hsa_circ_0005505 predicted targeting miRNAs, the circRNA-miRNA-mRNA network has been built (Figure 7). Then GO and KEGG pathway analysis of genes in hsa_circ_0005505-miRNA-mRNA network (Supplementary Table S6) have been performed in order to analyze the biological function of these genes. In the ontology analysis of molecular function, hsa_circ_0005505 correlated with genes such as MAP3K2, ACVRL1, CCNT2, RCC2, CTNND1, PTEN, PRKAG1, YWHAZ, CYLD, NR4A3, PDCD10, and mainly enriched in protein binding, DNA binding, RNA polymerase II core promoter proximal region sequence-specific DNA binding, zinc ion binding and sequence-specific DNA binding, respectively (Figure 8). In the biological process of gene oncology, hsa_circ_0005505 mainly correlated with ACVRL1, BTG2, CD86, PPP2CA, NF2, PTPN2 and so on and enriched in positive regulation of transcription, nervous system development, transcription, phosphatidylinositol-mediated signaling and regulation of transcription (Figure 8). In the ontology analysis of cellular component, genes such as CCNT2, CTNND1, DCAF8, MSANTD4, NR3C1, RBPJ, YY1, ELK4, PSMD correlated with hsa_circ_0005505 and mainly enriched in nucleus, nucleoplasm, PcG protein complex, cytoplasm, cytosol, respectively (Figure 8). The result of KEGG revealed that genes correlated with hsa_circ_0005505 enriched in Transcriptional misregulation in cancer, TGF-beta signaling pathway, MAPK signaling pathway, Melanoma and Ras signaling pathway (Figure 8). In brief, results of GO and KEGG pathway analysis suggested that, genes correlated with hsa_circ_0005505 may associated with cell proliferation and apoptosis.

### Knockdown of hsa_circ_0005505 Inhibits HBVSMCs Proliferation, Migration and Induces Apoptosis *In Virto*

**FIGURE 9:**
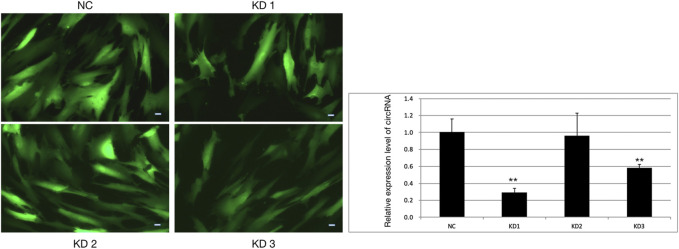
HBVSMCs infected by four kinds of lentivirus and the infection efficiency of lentivirus assessed by qPCR. NC = negative control, KD1 = lentivirus 1 used to reduce the expression of hsa_circ_0005505, KD2 = lentivirus 2 used to reduce the expression of hsa_circ_0005505, KD3 = lentivirus 3 used to reduce the expression of hsa_circ_0005505. Scale bar = 50 μm.

**FIGURE 10:**
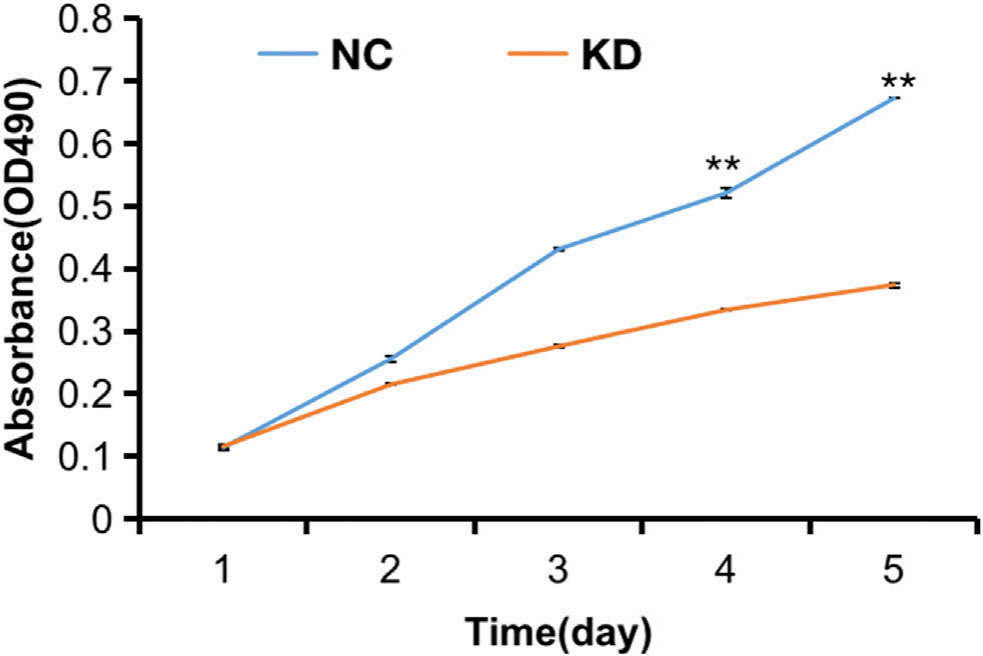
MTT assay of HBVSMCs transfected with hsa_circ_0005505 shRNAs or negative control were performed to evaluate cell viability ability. KD = knockdown of hsa_circ_0005505,NC = negative control. *p < 0.05, **p < 0.01, ***p < 0.001.

**FIGURE 11:**
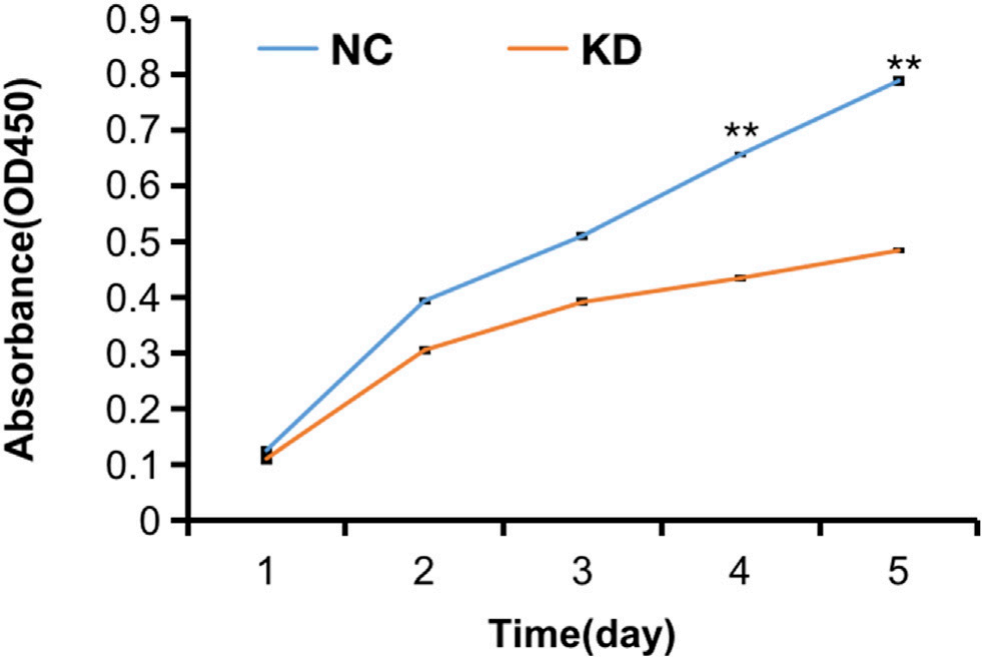
CCK8 assay of HBVSMCs transfected with hsa_circ_0005505 shRNAs or negative control were performed to evaluate cell proliferation ability. KD = knockdown of hsa_circ_0005505, NC = negative control. *p < 0.05, **p < 0.01, ***p < 0.001.

**FIGURE 12:**
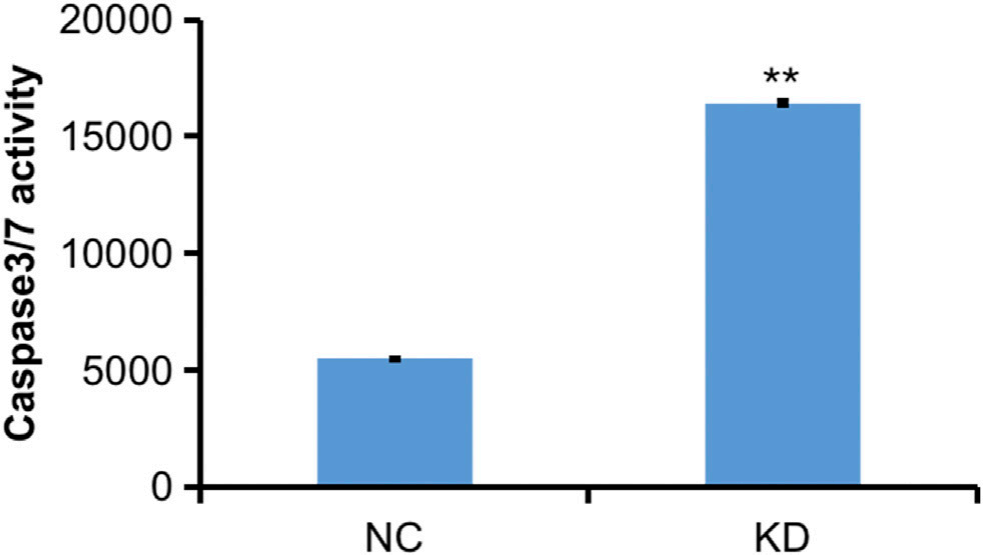
Caspase 3-7 analysis of HBVSMCs transfected with control or hsa_circ_0005505 shRNAs. KD = knockdown of hsa_circ_0005505, NC= negative control. *p < 0.05, **p < 0.01, ***p < 0.001.

**FIGURE 13:**
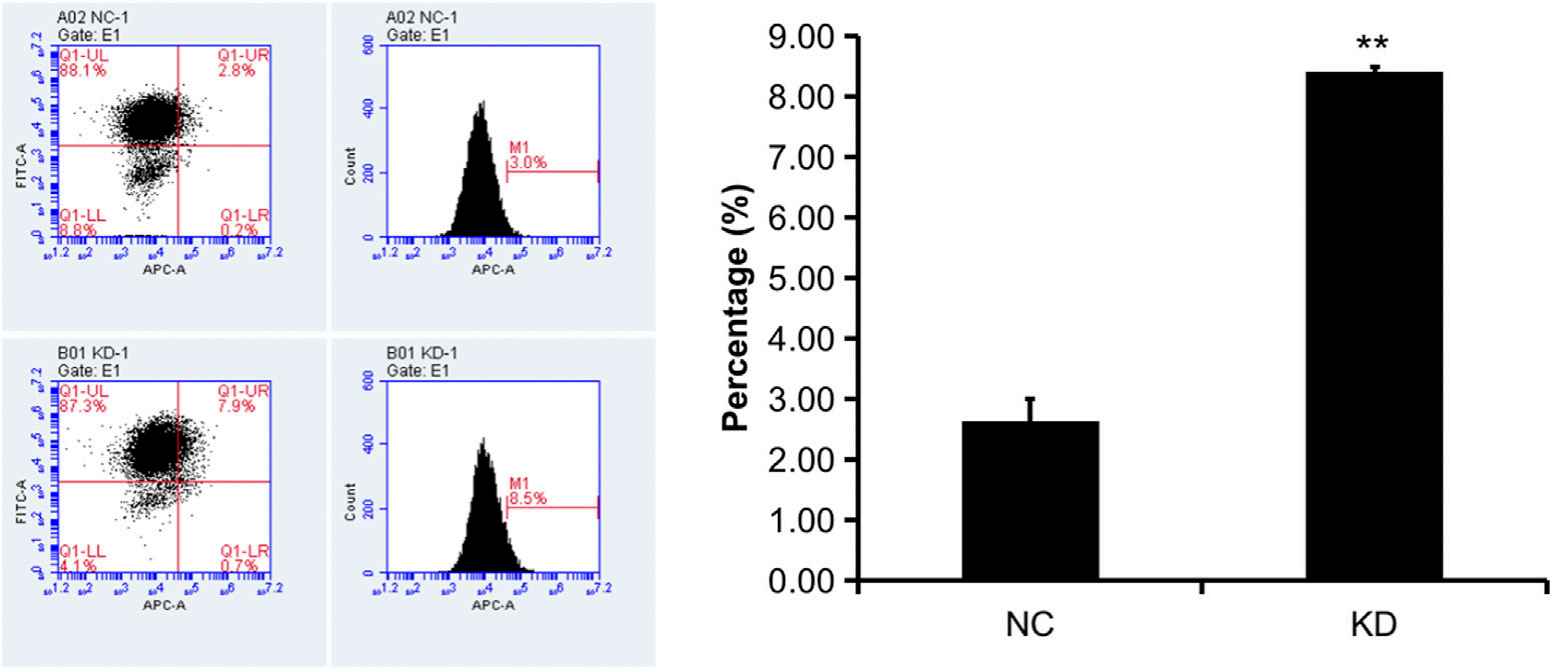
Flow cytometry apoptosis analysis of HBVSMCs transfected with control or hsa_circ_0005505 shRNAs. KD = knockdown of hsa_circ_0005505, NC = negative control. *p < 0.05, **p < 0.01, ***p < 0.001.

**FIGURE 14:**
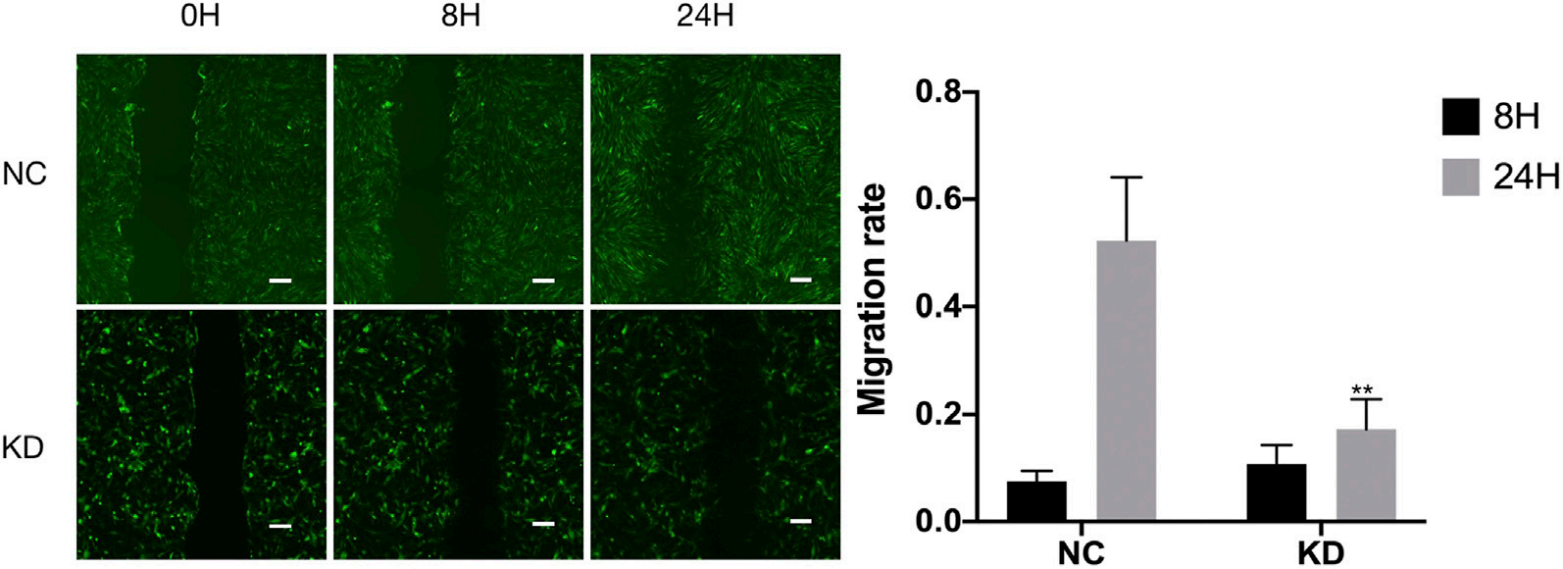
Wound-healing assay on hsa_circ_0005505 silenced HBVSMCs and negative control. The graph shows quantification of the residual wounded area 0, 8, and 24 h after scratch. Scale bar = 100 μm. KD = knockdown of hsa_circ_0005505, NC = negative control. *p < 0.05, **p < 0.01, ***p < 0.001.

**FIGURE 15:**
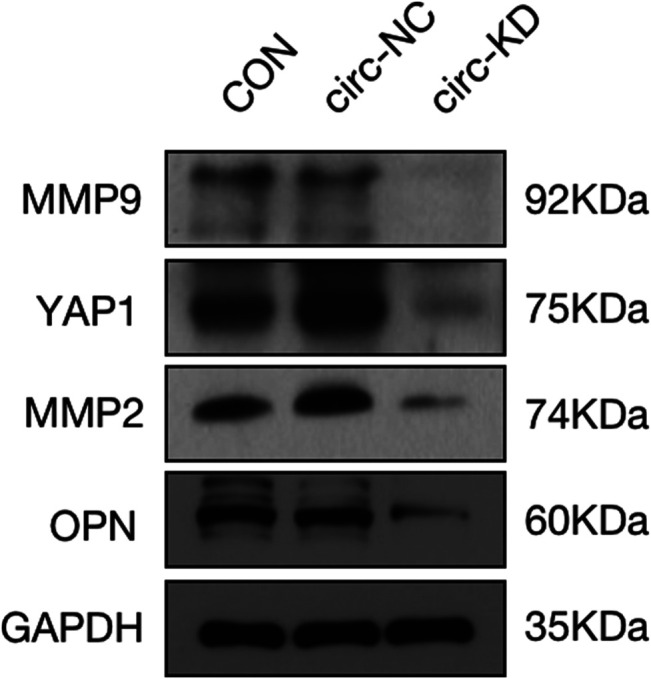
Protein expression level of MMP2, MMP9, YAP1, OPN and GAPDH assessed by western blot. GAPDH served as internal control. CON = control,KD = knockdown of hsa_circ_0005505, NC = negative control.

The pictures of infected HBVSMCs were shown in Figure 9. Then qPCR was performed to evaluate the transduction efficiency of three virus (Figure 9). Based on the result of qPCR, HBVSMCs infected by LV-circRNA-RNAi (74402-1) (KD1 in Figure 9) were selected for further experiments. Knockdown of hsa_circ_0005505 markedly inhibits HBVSMCs proliferation after transfected by shRNA according to the MTT assay (Figure 10) which was in consistent with the result of CCK8 assay (Figure 11). The effect of hsa_circ_0005505 on apoptosis of HBVSMCs was also analyzed. The result of apoptosis analysis suggested that knockdown of hsa_circ_0005505 significantly promotes HBVSMCs apoptosis (Figures 12, 13). Besides, the Wound-healing assay demonstrated that hsa_circ_0005505 silencing significantly impeded HBVSMCs migration (Figure 14). To further corroborate the effort of hsa_circ_0005505 on HBVSMC, western blot was performed subsequently. The results showed that, the inhibition of hsa_circ_0005505 reduced the protein level of known HBVSMC phenotype switch marker including OPN (Jiang et al., 2014), YAP1 (Xie et al., 2012) and also reduced the expression level of MMP2 and MMP9 (Figure 15).

## Discussion

The development of high-throughput sequencing, gene chip technology and bioinformatics over the last decade have largely improved our knowledge on circRNA. It is becoming increasingly clear that circRNAs play a crucial role in the pathological process of many kinds of cancers, such as colorectal cancer (Zeng et al., 2018), breast cancer (Liu et al., 2019), and hepatocellular carcinoma (Yu et al., 2018). However, whether circRNA participate in the pathological process of intracranial aneurysm is still unknown. In this study, we found hsa_circ_0005505 significantly upregulated in ruptured intracranial aneurysm tissues. Characteristics of hsa_circ_0005505 have been revealed and bioinformatical analysis of hsa_circ_0005505 also have been performed. Knockdown of hsa_circ_0005505 inhibits the proliferation, migration and induces apoptosis of HBVSMCs, which means hsa_circ_0005505 promotes the proliferation, migration of HBVSMCs but suppresses their apoptosis. Besides, the silencing of hsa_circ_0005505 reduce the protein level of OPN, YAP1, MMP2 and MMP9, in other words, hsa_cirx_0005505 promotes the expression of OPN, YAP1, MMP2 and MMP9. According to research about the formation and rupture of intracranial aneurysm, smooth muscle cell and it’s phenotypic modulation plays a significant role in these processes (Starke et al., 2014). With the influence of multiple stimuli, VSMCs were modulated from differentiated VSMC concerned with contraction to cells with a pro-inflammatory, pro-matrix remodeling phenotype characterized by the increased expression of OPN, YAP1, inflammatory factors and MMP, besides, both the proliferation and migration of VSMC can be promoted (Ishibashi et al., 2004; Kilic et al., 2005; Aoki et al., 2007; Aoki et al., 2010). Nakajima et al. (2000) suggested that VSMC phenotype modulation appeared to be more common in ruptured compared with unruptured aneurysms and appeared to be related to a remodeling of aneurysm wall and to a rupture mechanism. Following phenotypic modulation, both phenotypes of VSMC will loss and leading to aneurysm rupture eventually (Nakajima et al., 2000). These findings revealed that phenotype modulation of VSMC exists in the whole process of intracranial aneurysm formation and rupture. Based on these studies, we make a hypothesis that hsa_circ_0005505 may play a pivotal role in the phenotypic modulation of HBVSMCs.

Research about the role of circRNAs in the formation and rupture of intracranial aneurysm is still scarce. However, several circRNAs have been found to be related with VSMCs. Holdt et al. (2016) demonstrated that circ_ANRIL inhibits the proliferation and promotes the apoptosis of VSMCs and is associated with coronary artery disease. Hall et al. (2019) found circ_Lrp6 is a sponge for miR-145 and circ_Lrp6 hindered miR-145-mediated regulation of VSMC migration, proliferation, and differentiation, furthermore, the ratio of circ_Lrp6 bound to miR-145 versus unbound could play a role in vascular pathogenesis. CircRNAs mainly regulate gene transcription by acting as miRNA sponges, miRNAs binding with hsa_circ_0005505 have been revealed and we also have constructed the hsa_circ_0005505-miRNA-mRNA network. We will further explore the interaction of hsa_circ_0005505 with miRNAs and reveal the precise function of hsa_circ_0005505 in the pathological process of intracranial aneurysm.

Besides function as miRNA sponges to control gene transcription, circRNAs’ ability of encoding peptides or proteins also have been studied (Pamudurti et al., 2017; Yang et al., 2017). We have found that the ORF of hsa_circ_0005505 may encode PKC_like super family through bioinformatic analysis. PKC_like super family contains at least 11 isozymes and can be classified into three groups (Yamamoto et al., 2000). Existing studies demonstrate that PKC promotes the proliferation, migration and dedifferentiation of VSMCs (Rasmussen et al., 1984; Sobey and Faraci, 1998; Calabro et al., 2004; Ding et al., 2011) and this is in line with our results about the function of hsa_circ_0005505 in virto. During the pathological process of intracranial aneurysm, the VSMC can undergo phenotype modulation. With the stimulation of environment, VSMC can transform from a differentiated phenotype concerned mainly with contraction to an undifferentiated, pro-inflammatory, promatrix -remodeling phenotype (Starke et al., 2014). Furthermore, Zuniga et al. (2017) found that a member of PKC_like super family, PKC-epsilon, is a key mediator in resistin-induced inflammation by promoting the activation of NF-κB. All these findings suggest that PKC may play an important role in the formation and rupture of intracranial aneurysm and we’ll verify this hypothesis in our further study.

Our study also has some limitations. Firstly, the amount of our tissue sample is small. Our results need more intracranial aneurysm samples to testify. Second, due to the rare of aneurysm samples, some of our samples were stored in liquid nitrogen for several weeks, perhaps this would affect the amount of circRNAs. Lastly, the lack of studies about circRNAs in aneurysm make us can’t compare our results with others in order to improve our methods.

The above findings revealed an important role of hsa_circ_0005505 in the proliferation, migration and apoptosis of VSMCs and may take part in the phenotype modulation of VSMCs, indicating that hsa_circ_0005505 may associated with the pathological process of intracranial aneurysms.

## Data Availability

The datasets presented in this study can be found in online repositories. The names of the repository/repositories and accession number(s) can be found below: ArrayExpress E-MTAB-10683.
