# Supplementary material for: The Potential Role of hsa_circ_0005505 in the Rupture of Human Intracranial Aneurysm
Source: Front Mol Biosci. 2021 Jul 14;8:670691. doi: 10.3389/fmolb.2021.670691 (PMC8316638; doi:10.3389/fmolb.2021.670691)
Supplement: Supplementary file 4 [file DataSheet1.docx]

Supplementary

Supplementary Table 1:RNA quantification and quality assurance by NanoDrop ND-1000

| **Sample ID OD 260/280 OD 260/230 Conc Volume Quantity QC result**  **Ratio Ratio (ng/μL) (μL) (ng) Pass or Fail** |
| --- |
| RIA1 1.86 2.19 140.84 15 2112.60 Pass  RIA2 1.82 2.32 297.77 15 4466.55 Pass  RIA3 1.89 2.29 611.74 15 9176.10 Pass  RIA4 1.96 2.30 1034.73 20 20694.6 Pass  RIA5 1.84 2.13 136.64 15 2049.6 Pass  UIA1 1.83 2.38 311.20 15 4668.00 Pass  UIA2 1.91 2.18 625.76 15 9386.40 Pass  UIA3 1.90 2.34 619.68 60 37180.80 Pass  UIA4 1.88 2.21 231.37 15 3470.55 Pass  UIA5 1.92 2.30 560.15 30 16804.50 Pass |

*For spectrophotometer, the O.D. A260 /A280 ratio should be close to 2.0 for pure RNA (ratios between 1.8 and 2.1 are acceptable). The O.D. A260/A230 ratio should be more than 1.8.

Supplementary Table 2:Labeling Efficiency

| **Sample ID Dye Dye cRNA concentration Specific activity* Volume Total amount** **name pmol/μL (μg/μL) (pmol dye/μg cRNA) (μL) (μg)** |
| --- |
| RIA1 Cy3 10.68 0.54152 19.72226 10 5.4152  RIA2 Cy3 11.34 0.59888 18.93535 10 5.9888  RIA3 Cy3 11.38 0.57492 19.79406 10 5.7492  RIA4 Cy3 12.57 0.65978 19.05181 10 6.5978  RIA5 Cy3 12.48 0.64182 19.44470 10 6.4182  UIA1 Cy3 11.92 0.61466 19.39284 10 6.1466  UIA2 Cy3 12.01 0.63992 18.76797 10 6.3992  UIA3 Cy3 13.08 0.64829 20.17616 10 6.4829  UIA4 Cy3 11.14 0.5862 19.00375 10 5.862  UIA5 Cy3 13.13 0.7341 17.88585 10 7.341 |

*For two-color, if the yield is <825 ng and the specific activity is <8.0 pmol Cy3 or Cy5 per μg cRNA do not proceed to the hybridization step. Repeat cRNA preparation.

*For one-color, if the yield is <1.65 μg and the specific activity is <9.0 pmol Cy3 or Cy5 per μg cRNA do not proceed to the hybridization step. Repeat cRNA preparation.

Supplementary Table 3: qRT-PCR primer sequences

| Primer name | Primer F (5'-3') | Primer R(5'-3') |
| --- | --- | --- |
| hsa_circ_0005505 | CATTCACTACCTGCACAACGTTC | GCTGCTTGAAAGTCTCTCTGCT |
| hsa_circ_0001947 | AAACCCAGTGCAGCCAGTTC | AGTGCATCACCTTTGTTTGTTTC |
| hsa_circ_0043001 | ACTCAGAAGCAGAACAGAGTGATG | GGAACTCTCTCTGGGGTGACAT |
| hsa_circ_0064557 | GGAGTGCCCCCTTTCACAG | GCCTCGTTCAAATGATCCATAC |
| hsa_circ_0058514 | GCACATTTCAACAGTCATGCAG | TTTCACCCTGTGTGGTGGATT |

Supplementary Table 4:Significant differentially expressed circRNAs (FC≥1.5 and P≤0.05) .

Supplementary Table 5:MicroRNAs binding on hsa_circ_0005505.

Supplementary Table 6: Target genes of microRNAs binding on hsa_circ_0005505.
